# Evaluation of Flu A/B, SARS-CoV-2, and RSV Antigen Combo Rapid Test in Hospitalized Children Under Two Years of Age

**DOI:** 10.3390/diagnostics16060830

**Published:** 2026-03-11

**Authors:** Birhan Mulugeta, Dessalegn Fentahun, Dawit Hailu, Asmare Moges, Abiy Ayele Angelo, Getu Girmay, Abaysew Ayele, Tesfaye Gelanew

**Affiliations:** 1 Armauer Hansen Research Institute, Addis Ababa P.O. Box 1005, Ethiopia; brshmule12@gmail.com (B.M.); dessalegnfentahun.muche@gmail.com (D.F.); asmaremoges22@gmail.com (A.M.); abaysew.ayele@ahri.gov.et (A.A.); 2 Department of Immunology and Molecular Biology, School of Biomedical and Laboratory Sciences, College of Medicine and Health Sciences, University of Gondar, Gondar P.O. Box 196, Ethiopia; abiyuog12@gmail.com (A.A.A.); getugirmay2008@yahoo.com (G.G.)

**Keywords:** respiratory virus, RSV, influenza virus, rRT-qPCR, combined or multiplex RDT, children, nasopharyngeal swabs, sensitivity, specificity

## Abstract

**Background/Objectives**: Next to malaria, respiratory viruses, particularly respiratory syncytial virus (RSV), are responsible for the hospitalization and death of thousands of young children each year in sub-Saharan Africa. During peak seasons, conducting separate tests is time-consuming and distressing. This underscores the need for efficient, rapid multiplexed diagnostic tools. This study aimed to evaluate the clinical performance of a lateral flow assay (LFA) based antigen combo rapid diagnostic test (ML Ag Combo RDT, manufactured by MobiLab) that detects RSV, influenza viruses A and B (Flu A/B), and SARS-CoV-2. **Methods**: The Allplex panel 1 rRT-qPCR assay was used as a reference assay to evaluate the clinical performance of the LFA Ag Combo RDT in pediatric hospital settings. It was performed using 470 nasopharyngeal swab (NPS) specimens from hospitalized children under two years of age with respiratory symptoms. **Results**: Based on the comparative analysis of the testing results for 470 NPS, the ML Ag Combo RDT demonstrated high sensitivity, specificity, positive predictive value (PPV), and negative predictive value (NPV) of 90.06%, 98.38%, 93.67, and 97.39% for RSV, and 30%, 100%, 100%, and 95.43 for Flu A/B, respectively. Agreement with the Allplex panle1 1 rRT-qPCR was strong (κ = 0.90 for RSV) and moderate (κ = 0.45 for Flu A/B), with overall accuracies of 96.63% for RSV and 95.5 for Flu A/B. This was further supported by ROC analysis for aggregated data (RSV and, Flu A/B) with an AUC value of 0.925. As expected, in samples with high viral loads (Ct < 20), the Ag Combo RDT achieved 100% sensitivity for RSV and Flu A/B. Sensitivity declined slightly at lower viral loads (Ct > 35). **Conclusions**: The ML Ag Combo RDT demonstrates high specificity and diagnostic accuracy for the detection of RSV and Flu A/B in pediatric hospital settings where timely diagnosis is critical.

## 1. Introduction

Acute respiratory infections (ARIs) remain one of the leading causes of hospitalization and death among children under two years, especially in low- and middle-income countries [1,2]. Among the most common viral culprits are respiratory syncytial virus (RSV), influenza viruses A and B (Flu A/B), and SARS-CoV-2, all of which contribute significantly to this burden [1,3]. RSV, in particular, is known for causing severe lower respiratory tract infections (LRTIs) in infants and toddlers, often requiring emergency care or hospitalization that requires oxygen support [4,5].

In Africa, RSV plays a major role in the burden of LRTIs among children under five, particularly among children under two years of age [2]. Its impact on child mortality and morbidity in children under two years old is further fueled by the high prevalence of HIV among pediatric populations, which increases vulnerability to severe RSV infections [2]. Economic challenges across the continent also compound the issue, limiting access to timely diagnosis and treatment and placing additional strain on families and healthcare systems [6,7].

In Ethiopia, ARIs remain a major public health concern. A recent case–control study in Addis Ababa found RSV A, RSV B, and Flu A/B significantly associated with LRTIs in children, particularly in those under two years of age, with RSV showing the highest attributable fraction [8,9]. Despite this, the burden in children under two is underreported, and surveillance data is fragmented. Except for one study, most of these studies used CDC multiplex RT-PCR for diagnosing respiratory viruses, and sample testing was performed only at specialized referral centers [10,11].

This centralized, molecular-based approach creates a diagnostic gap at the point of care, especially in emergency departments, where timely decisions are critical [12]. Rapid antigen-based multiplex tests offer a practical alternative: they are portable, easy to use, and require no specialized equipment. Their use in pediatric emergency settings could improve clinical decision-making, reduce unnecessary antibiotic prescriptions, and help curb antimicrobial resistance [13,14].

Respiratory viral co-infections are commonly observed in pediatric populations and are associated with an increased risk of severe outcomes [15], including hospitalization and mortality, while also complicating clinical diagnosis. In Ethiopia, high seasonal circulation of respiratory viruses such as RSV and Flu A/B has been reported among children under five, with a significant proportion of co-infections identified through multiplex rRT-PCR assays [8,9,10,11]. Additionally, infections with SARS-CoV-2, Flu A/B, and RSV often present with similar symptoms [16]. This makes clinical differentiation challenging. This is especially true in resource-limited pediatric and emergency settings. In such settings, having access to validated multiplex rapid antigen detection tests that can identify and distinguish multiple respiratory viruses quickly is essential for making informed clinical decisions in tertiary care.

Although rRT-PCR-based point-of-care tests (POCTs) offer superior sensitivity and specificity [16,17,18], their use in low- and middle-income countries (LMICs) is limited due to the need for specialized equipment, trained personnel, and higher per-test costs. In contrast, lateral-flow rapid antigen tests are more practical for emergency departments and intensive care units in LMICs, as they are affordable, easy to use, and provide visually interpretable results in under 20 min. Importantly, antigen-based multiplex or combo rapid diagnostic tests (RDTs) that detect multiple respiratory pathogens from a single specimen can accelerate decisions regarding antiviral therapy, isolation protocols, and reduce unnecessary antibiotic use—ultimately improving targeted treatment [19,20]. These tests may also reduce the need for additional diagnostic procedures, including invasive and costly investigations such as chest radiography [20].

In this study, we evaluated the diagnostic performance of the MobiLab SARS-CoV-2, Flu A/B, and RSV Antigen Combo RDT, an immunochromatographic assay designed to detect and differentiate these viruses from a single nasopharyngeal sample. We analyzed 470 nasopharyngeal swabs (NPS) from hospitalized pediatric patients with suspected respiratory infections. Sensitivity, specificity, and clinical utility were assessed using the Allplex™ Respiratory Panel 1 multiplex rRT-qPCR as the reference standard. We also examined correlations with viral load (Ct values) and evaluated the test’s point-of-care utility in resource-limited hospital settings.

## 2. Materials and Methods

### 2.1. Study Design, Period, and Setting

This was a hospital-based cross-sectional study conducted at three government referral hospitals (ALERT Hospital, Yekatit 12 Hospital, and Zewditu Memorial Hospital), Addis Ababa, Ethiopia, between January 2024 and September 2025. The study was designed to evaluate the diagnostic sensitivity, specificity, accuracy, Positive Predictive Value (PPV), Negative Predictive Value (NPV) and concordance of the SARS-CoV-2, Influenza A/B and RSV Antigen Combo RDT (MobiLab Medical Innovatives, San Diego, CA, USA), hereinafter referred to as the ML Ag combo RDT against the Allplex™ Respiratory Panel 1 (Flu/RSV/Flu A subtyping) (Seegene Inc., Seoul, Republic of Korea), a reference standard, in line with US FDA diagnostic evaluation guidelines [21,22]. The reference assay, hereinafter referred to as the Allplex rRT-qPCR.

### 2.2. Ethical Consideration

The study received approval from the AHRI/ALERT Ethical Review Committee (Protocol number: PO/037/24).

### 2.3. Collection of Clinical Samples and Data

As proper sampling collection can have an effect on the quality of the specimen and thereby the accuracy of testing results, nurses and physicians selected as collectors received an hour of training on NPS collection from children and were provided with a video. NPS were collected twice from each participant by admitting nurses or pediatricians using sterile flocked swabs. One swab was placed in a dry tube for POCT using the ML Ag Combo RDT (MobiLab Medical Innovatives, Inc., San Diego, CA, USA). The second swab was placed in viral transport medium (VTM), frozen at −20 °C at the study hospital, and transported in a cold box to the Armauer Hansen Research Institute (AHRI) within two weeks. Upon arrival, samples were stored at −80 °C until molecular testing was performed using Allplex panel 1 rRT-qPCR (Seegene Inc., Seoul, Republic of Korea).

Clinical and demographic data were collected through structured interviews with parents or guardians, conducted by trained nurses or physicians. Data were entered into REDCap, a secure web-based platform for research data management.

### 2.4. Clinical Specimens Testing by Allplex Multiplex rRT-qPCR

#### 2.4.1. RNA Extraction

Viral RNA was extracted from a total of 474 nasopharyngeal swab (NPS) specimens, which had been collected in a VTM and stored at −20 °C prior to processing. An automated RNA extraction from each specimen was performed on the Bioer NPA-32P extraction instrument (Hangzhou Bioer Technology, Hangzhou, China) using the MagaBio plus virus RNA extraction Kit II (Cat No. BSC87S1E, Hangzhou, China) according to the manufacturer’s instructions, with 300 μL starting volume and 70 μL final eluted RNA volume. All RNA samples (*n* = 474) were then tested using rRT-qPCR in a pool of eight RNA samples for the presence of RSV A/B and influenza A/B.

#### 2.4.2. Pooled RNA Samples Testing

We used pooled RNA samples for testing because of the cost and shortage of the Allplex panel 1 rRT-qPCR assay. We assessed the sensitivity of pooled RNAs by mixing one known positive RNA sample with a high Ct value with seven RNA samples, each confirmed negative by rRT-qPCR. The pooled RNA was adjusted by adding more positives and fewer negatives until a pool of four positives and four negatives was achieved. This resulted in four pooled RNA samples, and we then tested them by rRT-qPCR per the manufacturer’s procedures. The testing results showed that the positivity of pooled RNA remained unaffected by the number of negative samples or higher Ct values (Table S1). This experiment suggests that our RNA pooling strategy did not reduce detection sensitivity with Allplex panel 1 rRT-qPCR, even at low viral loads. After this validation, all RNA samples (*n* = 474) were tested by rRT-qPCR in pools of up to eight RNA samples. If the pooled RNA sample tested positive, each RNA sample in the pool was then tested individually. If it tested negative, each sample in the pool was recorded as negative.

#### 2.4.3. rRT-qPCR Assay

Viral detection was performed using Allplex panel 1 rRT-qPCR according to the manufacturer’s instructions. This assay is a multiplex real-time one-step RT-PCR assay that permits simultaneous amplification and detection of target nucleic acids of Flu A, Flu B, RSV A, RSV B, and subtyping Flu A (Flu A-H1, Flu A-H3, Flu A-H1pdm09), and Internal Control (IC), following the manufacturer’s instructions.

Briefly, 8 µL of the extracted RNA(s) was combined with 17 µL of the Allplex™ panel 1 reaction master mix for quantitative multiple amplification, yielding a final reaction volume of 25 µL. Amplification and fluorescence detection was performed using the CFX96^TM^ Real-time PCR Detection System (Bio-Rad Laboratories, Hercules, CA, USA) under the following thermal cycling conditions: 1 cycle of reverse transcription at 50 °C for 20 min and, initial denaturation at 95 °C for 15 min, succeeded by 45 amplification cycles of 95 °C for 10 s, 60 °C for 1 min, and 72 °C for 10 s. The assay includes an exogenous internal control to monitor sample integrity and amplification efficiency.

#### 2.4.4. rRT-qPCR Analysis

Upon completion of the amplification run, CFX96^TM^ Dx v 3.1 was used to export the data to Seegene Viewer software Version 3.1 (Seegene Inc., Republic of Korea) for interpretation and data analysis, which applies predefined algorithms to assess curve morphology and signal strength, ensuring consistency across runs. Accordingly, a positive test result for Flu A, Flu B, RSV A, and RSV B was recorded when a clear exponential fluorescence curve crossed the Ct at or before 42 cycles. A negative test result was recorded when no amplification curve for the pathogen’s target genes crossed the threshold line, and the internal control was successfully amplified (Figure S1). Testing quality was assured by the inclusion of an exogenous internal control. The test result was recorded as invalid when neither the target genes nor the internal control was amplified. When invalid tests occurred, retesting was performed using a new RNA extraction from another aliquot of the original specimen to rule out technical failure during extraction or amplification. Positive results were classified into different categories based on the cycles of threshold (Ct) values to evaluate whether the sensitivity of the ML Ag Combo Rapid Test correlates with the Ct. The Ct values of rRT-qPCR are inversely proportional to the viral load; thus, a lower Ct value indicates a higher viral load.

### 2.5. Clinical Specimens Testing by the ML Ag Combo Test

A total of 476 NPS specimens were evaluated using the ML Ag combo Rapid Test, a lateral-flow immunochromatographic assay for the qualitative detection and differentiation of RSV, Flu A/B, and SARS-CoV-2 antigens in upper-respiratory specimens. Specifically, the assay targets the nucleoprotein (NP) antigen specific to Flu A, the NP antigen specific to Flu B, the nucleocapsid (N) protein of SARS-CoV-2, and both the fusion (F) protein and NP antigens specific to RSV.

Testing was performed according to the manufacturer’s instructions. Briefly, each NPS was inserted into the extraction tube containing approximately 500 µL of extraction buffer, rotated at least 10 times while pressing against the tube wall to release viral antigens, and then removed while squeezing the swab to recover as much liquid as possible. Three drops (≈60 µL) of the processed sample were dispensed into the sample well of the test cassette.

The assay was run at room temperature (15–30 °C), and the results were visually interpreted after 15 min (not later than 20 min). The presence of a colored band in the control (C) region confirmed test validity. A colored line in the designated region for RSV (T), Flu A/B (B), or SARS-CoV-2 (T) indicated a positive result for that pathogen. In contrast, the absence of any test line with a visible control line was considered negative. All tests were performed and interpreted independently by two authors (B.M. and D.F.) using unaided visual inspection. In cases of discordant interpretation, a third author (T.G.) served as the adjudicator.

### 2.6. Statistical Analysis

To assess the diagnostic agreement between the ML Ag combo test and the Allplex rRT-qPCR, two-by-two contingency tables were constructed for each target pathogen. From these, sensitivity, specificity, accuracy, PPV, and NPV were calculated, along with their corresponding two-sided 95% confidence intervals (CIs). Cohen’s kappa coefficient (κ) was computed to quantify the overall consistency of the diagnostic performance of the ML Ag Combo RDT beyond chance. Values between 0.0 and 0.20 were considered poor, values between 0.21 and 0.40 were considered fair, values between 0.41 and 0.60 were considered moderate, values between 0.61 and 0.80 were considered strong, and values between 0.81 and 1.00 were considered nearly perfect [23]. Additionally, the area under the curve (AUC) for the receiver operating characteristic (ROC) analysis was done. Its interpretation is classified as follows: unsatisfactory if AUC < 0.7, acceptable if 0.7 ≤ AUC < 0.8, excellent if 0.8 ≤ AUC < 0.9, and outstanding if AUC ≥ 0.9 [24].

More so, for specimens that tested positive by the Allplex panel 1 rRT-qPCR, the correlation between Ct values and the qualitative results of the ML Ag combo test was evaluated using Cohen’s kappa coefficient. All statistical analyses and visualizations were performed in Python (version 3) with pandas, scikit-learn, and matplotlib libraries, with a significance threshold set at *p* < 0.05.

## 3. Results

A total of 492 children with respiratory symptoms were recruited for this study, and pediatric nurses or clinicians collected NPS specimens. However, 16 children were excluded from the evaluation due to missing data. The median age of the qualified children (*n* = 476) was 9 months (IQR: 4–15 months). The majority of these (59.03%) children were males. All of them were hospitalized and symptomatic with respiratory illness.

Of the 476 NPS specimens tested using the ML Ag-Combo RDT, 150 tested positive for RSV, six tested positive for Flu A/B, two tested positive for SARS-CoV-2, and two had a mixed infection of RSV and SARS-CoV-2. The remaining 316 samples tested negative for all three targeted viruses. Of the 150 RSV-positive samples, 145 of them were confirmed by rRT-qPCR (κ = 0.90). Meanwhile, 21 out of the 316 RDT negatives tested positive by rRT-PCR.

**Figure 1:**
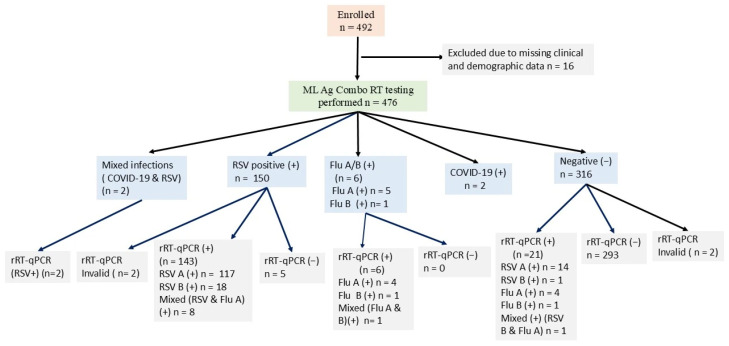
Final cohort for performance analysis: *n* = 470 (ML Ag-Combo RDT + rRT-qPCR results). *n* = 5 rRT-qPCR-negative by ML Ag Combo Rapid Diagnostic Test. ‘Negative’ group (*n* = 293): >62% symptomatic inpatients tested negative by both RDT and rRT-qPCR, possibly had infection with other respiratory pathogens. Abbreviations: Flu A, influenza virus A; Flu B, influenza virus B; RSV, respiratory syncytial virus; RSV A, respiratory syncytial virus A; RSV B, respiratory syncytial virus B; COVID-19, Coronavirus Disease 2019; ML Ag Combo RDT, MobiLab Antigen Combo Rapid Diagnostic Test; rRT-qPCR, real-time reverse transcriptase quantitative polymerase chain reaction; (+), positive; (−), negative.

As shown in Figure 1, of the 492 NPs, 16 were excluded due to missing data, and 2 were excluded because the Allplex panel 1 rRT-qPCR does not detect SARS-CoV-2. An additional 4 NPs samples were excluded because they yielded invalid rRT-qPCR test results. This left 470 NPs for ML Ag Combo RDT performance evaluation. Additionally, Figure 1 provides a visual summary of the workflow from recruitment to the final cohort for performance analysis, while Table S2 summarizes the two-by-two table test results by both assays and was utilized for performance analysis.

### 3.1. Mixed Infections

The Allplex rRT-qPCR assay identified ten cases (*n* = 10) of mixed infections among the 470 NPS included for ML Ag Combo RDT performance evaluation. Nine of these cases were identified as mixed infections with RSV and Flu A, while the remaining one was identified as a mixed infection with Flu A and Flu B. This finding aligns with the relative predominance of the two viruses in our cohort of hospitalized pediatric patients, where RSV was found to be predominant (~34%), followed by influenza viruses (4.4%). Among these mixed infections, the ML Ag Combo RDT classified eight cases as RSV mono-infections, while one case was identified as negative (Figures S1 and S2).

Confirmation of the two samples that had mixed infection with RSV and SARS-CoV-2 by RDT was not possible, as the Allplex™ panel 1 did not include primers and probes for SARS-CoV-2 detection. Nevertheless, both cases were confirmed as RSV-positive by Allplex panel 1 rRT-qPCR.

### 3.2. Clinical Performance of the ML Ag Como Rapid Test Using Allplex rRT-qPCR as a Reference

#### 3.2.1. ML Ag Combo RDT Sensitivity Depending on the Ct Values

As expected, the ML Ag Combo RDT demonstrated 100% sensitivity in NPs samples with Ct values below 20 for RSV and Flu A/B. It also showed high sensitivity at Ct values below 25, with 96.9% specificity for RSV and 100% specificity for Flu A/B. However, at Ct values below 42, its sensitivity decreased to 90.1% for RSV and 71.4% for Flu A/B (Table 1).

**Figure 2:**
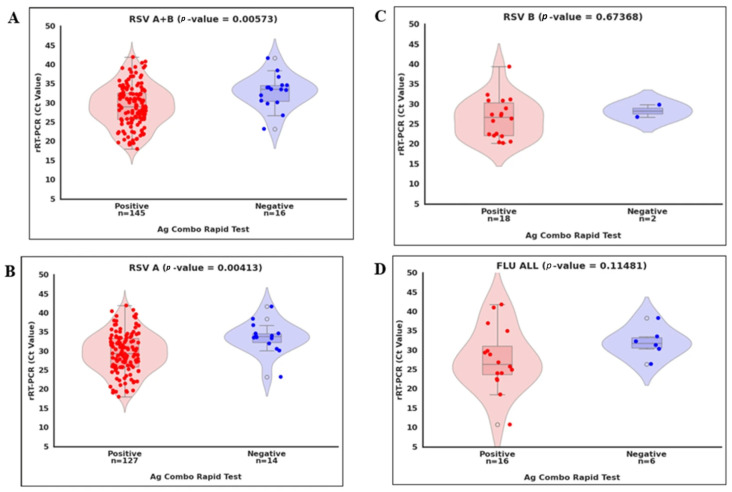
Positivity of the ML Ag Combo Rapid Test according to the cycle threshold (Ct) values of the corresponding Allplex rRT-qPCR test for the same virus. (**A**) All RSV rRT-qPCR positive samples (*n* = 161; (**B**) RSV A rRT-qPCR positive samples (*n* = 141); (**C**) RSV B rRT-qPCR positive samples (*n* = 20), and (**D**) All Flu rRT-qPCR positive samples (*n* = 21). The dot plots show the positive (left) and negative (right) results for Allplex rRT-qPCR. Abbreviations: Ct, cycle threshold; Flu ALL, Influenza viruses (Flu A and Flu B).

As shown in Figure 2, we compared viral loads (Ct values) by categorizing RT-PCR-positive samples into two groups: concordant (positive by the ML Ag Combo RDT) and discordant (negative). We also compared the viral load difference between concordance and discordance per virus. We found a statistically significant difference in Ct values between concordant and discordant RSV A + B and RSV A cases (*p* < 0.01; Figure 2A,B and Table 1). However, no significant difference was detected for influenza A/BB and RSV B (*p* > 0.05; Figure 2C,D), likely due to the limited number of positive cases identified for these viruses. Overall, these observations suggest that discordant results likely occurred near the detection limit of the ML Ag Combo RDT.

#### 3.2.2. ML Ag Combo RDT Showed High Performance Across Different Metrics in Pediatric Hospital Settings

We evaluated the performance of the ML Ag Combo RDT in detecting RSV (types A and B) and influenza virses (types A or B) among Ethiopian children under 2 with ILI, using a 21% prevalence of RSV [10]. Performance metrics included were sensitivity, specificity, PPV, NPV, overall accuracy, and agreement with reference methods. Cohen’s kappa (κ) was used to assess these metrics. Based on 470 NPS test results available for head-to-head comparison, the ML Ag Combo RDT demonstrated higher sensitivity for RSV (90.06%; 95% CI: 84.36–94.21%) than for Flu A/B (30% CI: 11.89–54.28%). However, the overall accuracy was similar for both targets: 96.63% (95% CI: 94.58–98.07%) for RSV and 95.52 (95% CI: 93.24–97.20% for Flu A/B (Table 2).

The ML Ag Combo RDT showed a slightly higher positive predictive PPV for influenza A/B (100%; 95% CI: 54.07–100%) than for RSV (93.67%; 95% CI: 86.10–97.25). Conversely, the NPV was higher for RSV (97.39%; 95% CI: 95.90–98.34%) than for Flu A/B (95.43%; 95% CI: 94.01–96.53%) (Table 2).

The ML Ag Combo RDT and the reference rRT-qPCR assay showed strong agreement (Cohen’s κ = 0.90 [95% CI: 0.86–0.94] for RSV and moderate agreement 0.45 [95% CI: 0.214–0.69%] for Flu A/B, Table 2). These results suggest that the ML Ag Combo RDT is a reliable tool for detecting both viruses and shows strong concordance with the reference method for RSV. The high performance observed for RSV could be due to the fact that the assay detects two RSV antigens, the F and N proteins, which likely enhances its sensitivity, even in samples with lower concentrations.

**Figure 3:**
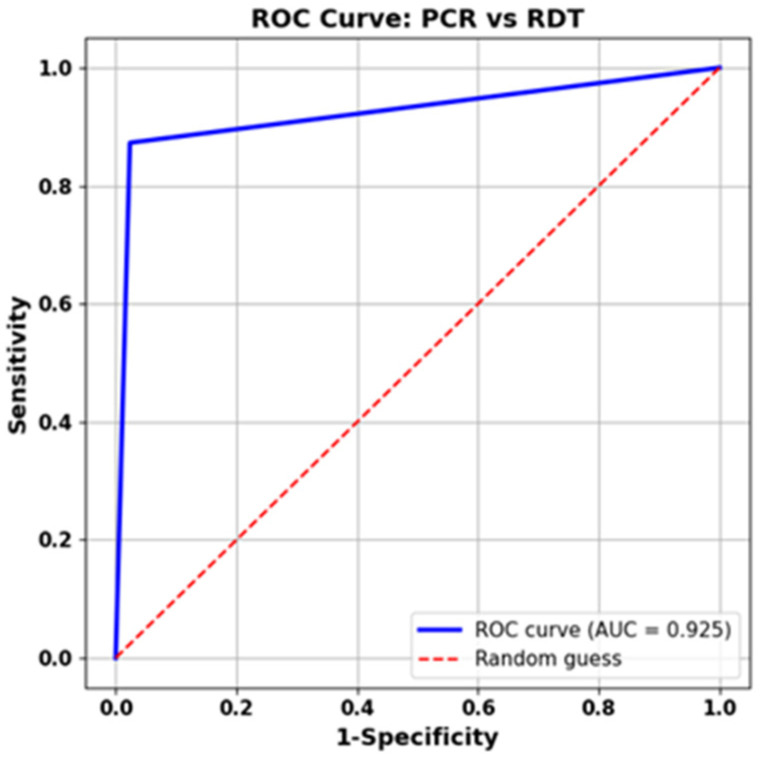
ROC analysis to evaluate the diagnostic value of the ML Ag Combo RDT for overall detection of RSV and Influenza viruses.

Furthermore, as shown in Figure 3, we performed ROC curve analysis on the aggregated dataset, combining samples from all viruses, excluding SARS-CoV-2, as our reference, as rRT-qPCR did not detect it. The determined AUC for the ML Ag Combo RDT was 0.925, suggesting outstanding accuracy in detecting RSV and influenza virus infections.

We used all clinical specimens that tested negative by Allplex panel 1rRT-qPCR as true negatives (*n* = 298) to calculate the specificity of the ML Ag Combo RT for Flu A, Flu B, and RSV (*n* = 470). The ML Ag Combo RDT had high specificity for RSV (98.38%, CI: 96.26–99.47%) and 100% for Flu A/B (CI: 99.18–100%) (Table 1 and Table 2). Five samples that were identified as RSV by ML Ag Combo RDT were found to be negative by Allpex panel 1 rRT-qPCR. To rule out the possibility of RNA extraction failure, these samples were retested using freshly extracted RNA from the original sample. None of the retests were positive by rRT-qPCR, and all had valid internal control results (Figure 1). These five samples may represent false positives or post-infectious antigen persistence, which affects specificity. If the latter were the case, the RSV specificity could be even higher than 98.0%.

## 4. Discussion

In Ethiopia, respiratory viruses such as SARS-CoV-2, RSV, and Flu A/B are co-circulating. During seasonal peak transmission, these viruses lead to hospitalization of children, particularly infants and young children under two years old with respiratory symptoms [9,25,26]. Performing multiple tests to identify and distinguish the virus(es) responsible for infection is time-consuming for healthcare workers and uncomfortable for children. Rapid multiplexed, or Combo antigen detection tests can offer a more efficient alternative by detecting multiple viral pathogens from a single sample, guiding clinical management, and implementing effective control measures [27,28]. This highlights the urgent need for validated multiplex or combo POCTs that can detect and differentiate these pathogens within two hours using just one clinical specimen. Such tools, especially in resource-limited settings like Ethiopia, can substantially improve the quality of pediatric care services by streamlining patient triage, optimizing bed usage, and reducing the risk of hospital-acquired infections [29]. When test results from these RDTs are combined with POCTs that detect host biomarkers such as CRP and myxovirus resistance protein A (MxA), it can significantly reduce antibiotic misuse [30].

This study evaluated the clinical performance of the ML Ag Combo RDT, a combined lateral flow assay designed to detect SARS-CoV-2, Flu A/B, and RSV. The ML Ag Combo RDT’s performance was evaluated against the Allplex panel 1 rRT-qPCR assay, the reference standard capable of simultaneously detecting and differentiating RSV (RSV A and RSV B) and Flu A/B. Based on 470 NPS specimens with valid test results for both assays, the ML Ag Combo RDT demonstrated high sensitivity for RSV (90.06%) and moderate sensitivity for Flu A/B (30%) at a Ct cutoff of 42, as defined by the manufacturer. Restricting the analysis to samples with Ct values below 20, which indicates high viral loads, resulted in 100% sensitivity for both RSV and Flu A/B. These results suggest that the sensitivity of the ML Ag Combo RDT is closely related to viral load. The RDT also demonstrated high specificity, reaching 100% for Flu A/B and 98.3% for RSV. There was strong overall agreement with the reference rRT-qPCR assay, with Cohen’s kappa (κ) values of 0.90 for RSV and moderate agreement with Cohen’s kappa (κ) values of 0.45 for Flu A/B, indicating excellent concordance.

The sensitivity and specificity performance metrics from our evaluation suggest that the ML Ag Combo RDT is a reliable tool for detecting RSV and Flu A/B, with strong concordance with the reference rRT-qPCR assay for RSV. Its sensitivity was found to be better for RSV than Flu A/B. Perhaps relatively higher sensitivity for RSV because it targets two antigens (the F protein and the N protein) for RSV, while for Flu A/B, it targets only the N protein. Interestingly, unlike the ML Ag Combo RDT, most commercial RSV RDTs target either the F protein or the N protein [28]. For example, the BinaxNOW™ RSV by Abbott detects the F protein, while the BD Veritor™ RSV by Becton Dickinson detects the N protein. These antigens may be expressed at different stages of RSV infection, allowing for detection across varying viral loads and time points.

The performance of the ML Ag Combo RDT for Flu A/B meets or exceeds the performance metrics previously reported for two commercially available combo antigen tests: the SARS-CoV-2/Flu A/B/RSV Antigen Combo Rapid Test (Alltest) and the SARS-CoV-2/Flu A + B/RSV Antigen Rapid Test (Qingdao HighTop) [16]. Both assays demonstrated high specificity, ranging from 99.48% to 100%, but sensitivity varied. The Alltest had sensitivities of 73.0% for Flu A and 44.4% for RSV in samples with Ct values < 25. The ML Ag Combo RDT demonstrated higher sensitivity under similar Ct values < 25—100% for Flu A and 96.9% for RSV. The Qingdao HighTop test reported sensitivities of 85% for Flu A and 100% for RSV in samples with Ct < 25. The ML Ag Combo RDT exhibits superior sensitivity compared to the Alltest and is comparable to, if not better than, the Qingdao HighTop assay.

The present evaluated test achieved 100% sensitivity for RSV NPS samples with higher viral loads (Ct < 20), mirroring the findings of Widyasari et al. [31], who reported near-perfect RSV detection in high viral load specimens using the STANDARD QCOVID/FLU Ag Combo test.

The ML Ag Combo RDT’s performance, particularly its sensitivity and specificity, was found to be either superior or non-inferior to other antigen-based POCTs for respiratory virus detection listed in the review paper by Basile and colleagues [28]. The BD Directigen™ EZ Flu A/B, BD Veritor™ Flu A/B, BD Veritor™ RSV (Becton Dickinson, Franklin Lakes, NJ, USA), and the D Bioline Influenza Ag (Standard Diagnostics Inc., Gyeonggi, Republic of Korea) are the most widely used assays for this purpose. The lateral flow immunochromatographic platforms of these tests are similar to those of the ML Ag Combo RDT, further underscoring its comparative strength within this diagnostic category.

Our findings are consistent with those of Rosenblatt and colleagues [31], who found similar κ values, as we found strong agreement between the ML Ag Combo RDT and the reference laboratory-based Allplex panel 1 rRT-qPCR assay, with κ values of 0.90 for RSV and 0.45 for Flu A/B. This is further corroborated by AUC 0.925. This high level of agreement with our laboratory-based reference molecular method supports the use of the ML Ag Combo RDT as a POCT for pediatric patients presenting with respiratory symptoms.

Unlike our study, the Fluorecare^®^ SARS-CoV-2 and Flu A/B and RSV rapid antigen combo test demonstrated the highest sensitivity for Flu A (80.8%) and the lowest sensitivity for RSV (41.5%) [32]. However, unlike our study, this study evaluated specimens from symptomatic children and adults presenting to the emergency department with flu-like symptoms. Additionally, a UK study reported slightly lower sensitivity (67.0%) for influenza using a multiplex LFA in adults with flu-like symptoms [29]. The differences in the populations studied likely contributed to the variation in the performance of the tests between these studies and ours. Symptomatic children are more likely to have higher viral loads, which could improve the ML Ag Combo RDT’s sensitivity. The enhanced sensitivity may also be linked to the kit design and the nature of RDT design (combo vs. multiplex). Combo antigen detection tests, like ours, may offer superior sensitivity compared to multiplexed platforms that target the same number of viral pathogens. Unlike multiplex platforms, which share reagents across multiple targets, combo formats have no competition for reagents or binding sites; each pathogen is detected in a dedicated reaction zone (Figure S2).

The accuracy of the variety of commercial multiplex or combo antigen RDTs for detection of respiratory viruses such SARS-CoV-2, influenza and RSV is dependent on the tested populations (symptomatic vs. asymptomatic; age of subject: children or vs. adults), timing of sample collection from onset of symptoms, and chosen sample material, such as, nasal, nasopharyngeal, oropharyngeal, and throat swabs are used [28,33,34]. For example, lower sensitivity for RSV was observed when throat swab was used instead of a nasal, nasopharyngeal, or trachea secretes [35]. NPS was found to be a good matrix for the detection of RSV and influenza virus in children using the BD Veritor™ System Rapid Test [36]. In our study, only NPS were analyzed, and all of them were collected from hospitalized children with respiratory symptoms, a population likely to have higher viral loads. As such, the performance of the ML Ag Combo RDT may be overestimated when extrapolated to non-hospitalized or mildly symptomatic children in community settings, as well as in hospitalized adult subjects with respiratory symptoms. This concern is echoed by Caldwell and colleagues [12], who noted that antigen test sensitivity tends to decline in outpatient or low-viral-load populations.

Notably, over 62% of symptomatic pediatric inpatients tested negative for SARS-CoV-2, influenza viruses, and RSV (Figure 2), highlighting the need for host biomarkers such as myxovirus resistance protein A (MxA) and C-reactive protein (CRP)-based point-of-care tests [37] to guide antibiotic use and clinical triage. Similar observations were reported by Lim et al. [20], who emphasized the need for broader diagnostic panels or follow-up molecular testing in cases with high clinical suspicion but negative antigen results. We are currently evaluating the utility of the Fluorescence immunoassay (FIA) MxA assay and FIA CRP in our ongoing studies.

The 10 mixed infections (9 RSV plus Flu A and 1 Flu A plus Flu B) were detected by rRT-qPCR. In contrast, the Ag Combo Rapid Test misclassified 8 of these as mono-infections and reported 1 as negative, indicating its limited ability to identify mixed respiratory infections compared to multiplex rRT-qPCR. However, this limitation is not unique to the Ag Combo Rapid Test. Multiplex rRT-qPCR assays have higher sensitivity for detecting co-infections than antigen-based RDTs but require trained personnel and are typically conducted in laboratory settings. Conversely, multiplex or combined antigen RDTs are faster and easier to use at the point of care diagnostic test, but may miss co-infections. Since co-infections are common in pediatric populations and can affect disease severity, treatment decisions, and outcomes, choosing between antigen-based RDTs and multiplexed rRT-qPCR assays should consider balancing robustness, turnaround time, and detection capacity. Most importantly, in resource-limited settings, where multiplex rRT-qPCR is unavailable, and treatment is usually based on syndromic diagnosis, the clinical use of the Ag Combo Rapid Test, along with clinical assessment, should be considered and valued.

## 5. Study Strengths and Limitations

The study demonstrates several key strengths. First, it is the first in Ethiopia to validate a Ag Combo RDT for detecting RSV, Flu A/B, and SARS-CoV-2 from a single sample. Its ability to detect multiple pathogens with high accuracy supports a critical shift from syndromic management to diagnosis-driven, pathogen-specific treatment. Second, the sample size—particularly for RSV—was sufficiently large to ensure reliable analysis and strengthen the validity of the findings. Third, the study was conducted with strong methodological and analytical rigor, including the use of a well-established reference multiplex rRT-qPCR assay, which further reinforces the credibility of the results.

However, our study had limitations as well. One important limitation was the lack of a third diagnostic method to settle disagreements between the ML Ag Combo RDT and rRT-qPCR tests. For example, five samples that tested positive with the ML Ag Combo RDT tested negative with rRT-qPCR. These discordant results may indicate true positives that were missed by rRT-PCR, particularly since antigen-based tests can detect residual viral proteins even after RNA has cleared. This is particularly relevant in pediatric patients who may have recently suffered RSV infections, but the virus has cleared to the level not to be detected by rRT-qPCR.

Another limitation was the underrepresentation of RSV B and influenza viruses, especially Flu B, which likely stems from our study population of hospitalized children under two years of age presenting with flu-like symptoms. Flu B circulates more widely among older children and adolescents, so its underrepresentation may reflect our age-specific sampling. Had we included older children, the prevalence of influenza, particularly Flu B, might have been higher. Additionally, RSV A has been more frequently associated with severe disease in young children than RSV B [38].

A third limitation is that our samples came exclusively from symptomatic pediatric inpatients. Antigen tests generally show higher sensitivity in symptomatic children, especially early in the illness [36]. Consequently, the performance of the ML Ag Combo RDT in our study may not reflect its accuracy in asymptomatic children, where sensitivity is likely lower.

A fourth limitation of this study is that the ML Ag Combo RDT does not detect other common respiratory viruses in children, such as rhinoviruses and adenoviruses [15,39]. Empirical treatment with antimicrobials could not be ruled out in positive cases unless test results are paired with host biomarker-based POCTs that distinguish viral from bacterial infections.

The fifth limitation is that our reference standard, the Allplex™ Respiratory Panel 1, does not detect SARS-CoV-2. Consequently, no performance evaluation (sensitivity, specificity, PPV, NPV) for the SARS-CoV-2 component of the RDT was provided.

In this study, the combined antigen RDT demonstrated a comparatively limited capacity to detect mixed RSV and Flu A/B infections compared to rRT-qPCR. Consequently, in settings with a high prevalence of co-infections where multiplex rRT-qPCR assays are not readily accessible, the clinical utility of the ML Ag Combo RDT should be appraised with caution.

Lastly, due to funding constraints, we did not conduct follow-up on treatment types and outcomes, limiting our ability to assess the impact of the ML Ag Combo RDT on antibiotic misuse.

## 6. Conclusions

The ML Ag Combo RDT demonstrates high specificity and diagnostic accuracy for RSV and Flu A/B detection in pediatric hospital settings (90.06% sensitivity; 98.38% specificity for RSV). Further studies with larger and more diverse populations, including asymptomatic and outpatient cases, are needed to confirm its broader clinical applicability.

## Figures and Tables

**Table 1:** Sensitivity and specificity of ML Ag Combo Rapid Test in Allplex rRT-qPCR-positive samples with different Ct values (*n* = 470 NPS samples).

Ct ^a^ (N Gene)	RSV	Flu A/B
<20	(7/7) 100%	(1/1) 100%
<25	(31/32) 96.9%	(6/6) 100%
<30	(78/81) 96.3%	(11/12) 91.6%
<35	(125/138) 90.6%	(12/17) 70.6%
<42	(145/161) 90.1%	(15/21) 71.4%
20–25	(24/25) 96%	(5/5) 100%
25–30	(47/49) 95.9%	(5/6) 83.3%
30–35	(46/56) 82.1%	(1/5) 20.0%
35–42	86.9% (20/23)	(3/4) 75.0%
Mean Ct (<42) [95% CI]	29.6% (28.7–30.4)	29.7% (26.8–32.6)
Neg. with rRT-qPCR [95% CI]	98.3% (96.13–99.45)	100% (98.78–100)

^a^ Abbreviations: Ct, cycle threshold; CI, confidence interval; RSV, Respiratory Syncytial Virus; rRT-qPCR, real-time reverse-transcription quantitative polymerase chain reaction; neg, tested negative for rRT-qPCR.

**Table 2:** Performance characteristics of the ML Ag Combo Rapid Test against Allplex rRT-qPCR, as a reference test for the detection of RSV (*n* = 470).

	RSV	95% CI ^1^	Flu A/B	95% CI
Sensitivity	90.06%	84.36–94.21%	30.0%	11.89–54.28%
Specificity	98.38%	96.26–99.47%	100%	99.18–100%
PPV	93.67%	85.10–97.25%	100%	54.07–100%
NPV	97.39%	95.90–98.34%	95.43%	94.01–96.53%
Accuracy	96.63%	94.58–98.07%	95.52%	93.24–97.20%
Cohen’s κ	0.90	0.86–0.94	0.45	0.214–0.69%

^1^ The calculation assumed that the prevalence of RSV-hospitalized children is 21% [25]. Abbreviations: PPV, positive predictive value; NPV, negative predictive value; CI, confidence interval.

## Data Availability

The data presented in this study are available on request from the corresponding author due to patients’ privacy.

## Supplementary Materials

The following supporting information can be downloaded at: https://www.mdpi.com/article/10.3390/diagnostics16060830/s1, Figure S1: rRT-qPCR graph showing Ct values for selected specimen samples; Figure S2: Photographs of the ML Ag Combo Rapid Test cassettes; Table S1: Sensitivity of Pooling of eight RNA samples with different proportions of known positive and negative RNA samples and their corresponding Ct values.; Table S2: Two-by-Two Table for Test comparison between ML Ag Combo Rapid Test and Allplex rRT-qPCR.

## Abbreviations

The following abbreviations are used in this manuscript:

AHRI	Armauer Hansen Research Institute
ARI	Acute Respiratory Infections
AUC	Arear Under Curve
CDC	Center for Disease Control
CI	Confidence Interval
COVID-19	Coronavirus Disease 2019
CRP	C-Reactive Protein
CT	Cycle Threshold
FIA	Fluorescence immunoassay
ILI	Influenza-like Illness
IQR	Inter Quartile Range
LFA	Lateral Flow Assay
LRTI	Lower Respiratory Tract Infection
ML	MobiLab
MxA	Myxovirus Resistance Protein A
NP	Nucleoprotein
NPS	Nasopharyngeal Swab
NPV	Negative Predictive Value
POCT	Point-of-care Test
PPV	Positive Predictive Value
RDT	Rapid Diagnostic Test
RNA	Ribo Nucleic Acid
RSV	Respiratory Syncytial Virus
Flu A	Influenza virus A
Flu B	Influenza virus B
RSV A	Respiratory syncytial virus A
RSV B	respiratory syncytial virus B
ROC	Receiver Operating Characteristics
rRT-qPCR	Real-Time Reverse Transcription Quantitative Polymerase Chain Reaction
SARS-CoV-2	Severe Acute Respiratory Syndrome-Coronavirus 2
VTM	Viral Transport Medium

## References

[1] Wang X., Li Y., Deloria-Knoll M., Madhi S.A., Cohen C., Arguelles V.L., Basnet S., Bassat Q., Brooks W.A., Echavarria M. (2021). Global burden of acute lower respiratory infection associated with human parainfluenza virus in children younger than 5 years for 2018: A systematic review and meta-analysis. Lancet Glob. Health.

[2] Robertson S.E., Roca A., Alonso P., Simoes E.A.F., Kartasasmita C.B., Olaleye D.O., Odaibo G.N., Collinson M., Venter M., Zhu Y. (2004). Respiratory syncytial virus infection: Denominator-based studies in Indonesia, Mozambique, Nigeria and South Africa. Bull. World Health Organ..

[3] 2023 Respiratory Virus Response—NSSP Emergency Department Visits—COVID-19, Flu, RSV, Combined|Data|Centers for Disease Control and Prevention. https://data.cdc.gov/Public-Health-Surveillance/2023-Respiratory-Virus-Response-NSSP-Emergency-Dep/vutn-jzwm/about_data.

[4] Langley J.M., Bianco V., Domachowske J.B., Madhi S.A., Stoszek S.K., Zaman K., Bueso A., Ceballos A., Cousin L., D’Andrea U. (2022). Incidence of Respiratory Syncytial Virus Lower Respiratory Tract Infections During the First 2 Years of Life: A Prospective Study Across Diverse Global Settings. J. Infect. Dis..

[5] Mazur N.I., Caballero M.T., Nunes M.C. (2024). Severe respiratory syncytial virus infection in children: Burden, management, and emerging therapies. Lancet.

[6] Moyes J., Tempia S., Walaza S., McMorrow M.L., Treurnicht F., Wolter N., von Gottberg A., Kahn K., Cohen A.L., Dawood H. (2023). The economic burden of RSV-associated illness in children aged <5 years, South Africa 2011–2016. BMC Med..

[7] Rave N., Pecenka C., Shaaban F.L., Bont L.J., RSV GOLD III—Health Economics Study Group (2025). Estimating the economic burden of respiratory syncytial virus infection among children <2 years old receiving care in Maputo, Mozambique. J. Glob. Health.

[8] Tayachew A., Teka G., Gebeyehu A., Shure W., Biru M., Chekol L., Berkessa T., Tigabu E., Gizachew L., Agune A. (2024). Prevalence of respiratory syncytial virus infection and associated factors in children aged under five years with severe acute respiratory illness and influenza-like illness in Ethiopia. IJID Reg..

[9] Wadilo F., Feleke A., Gebre M., Mihret W., Seyoum T., Melaku K., Howe R., Mulu A., Mihret A. (2023). Viral etiologies of lower respiratory tract infections in children <5 years of age in Addis Ababa, Ethiopia: A prospective case–control study. Virol. J..

[10] Tayachew A., Mekuria Z., Shure W., Arimide D.A., Gebeyehu A., Berkesa T., Gonta M., Teka G., Kebede M., Melese D. (2025). Epidemiology of respiratory syncytial virus and its subtypes among cases of influenza like illness and severe acute respiratory infection: Findings from nationwide sentinel surveillance in Ethiopia. BMC Infect. Dis..

[11] Shure W., Tayachew A., Berkessa T., Teka G., Biru M., Gebeyehu A., Woldeab A., Tadesse M., Gonta M., Agune A. (2024). SARS-CoV-2 co-detection with influenza and human respiratory syncytial virus in Ethiopia: Findings from the severe acute respiratory illness (SARI) and influenza-like illness (ILI) sentinel surveillance, January 01, 2021, to June 30, 2022. PLoS Glob. Public Health.

[12] Caldwell J.M., Espinosa C.M., Banerjee R., Domachowske J.B. (2025). Rapid diagnosis of acute pediatric respiratory infections with Point-of-Care and multiplex molecular testing. Infection.

[13] Jeong H.W., Heo J.Y., Park J.S., Kim W.J. (2014). Effect of the Influenza Virus Rapid Antigen Test on a Physician’s Decision to Prescribe Antibiotics and on Patient Length of Stay in the Emergency Department. PLoS ONE.

[14] Çelik M., Polat M.R., Avkan-Oğuz V. (2024). Diagnostic utility of rapid antigen testing as point-of-care test for influenza and other respiratory viruses in patients with acute respiratory illness. Diagn. Microbiol. Infect. Dis..

[15] Swets M.C., Russell C.D., Harrison E.M., Docherty A.B., Lone N., Girvan M., Hardwick H.E., Visser L.G., Openshaw P.J.M., Groeneveld G.H. (2022). SARS-CoV-2 co-infection with influenza viruses, respiratory syncytial virus, or adenoviruses. Lancet.

[16] Clark T.W., Lindsley K., Wigmosta T.B., Bhagat A., Hemmert R.B., Uyei J., Timbrook T.T. (2023). Rapid multiplex PCR for respiratory viruses reduces time to result and improves clinical care: Results of a systematic review and meta-analysis. J. Infect..

[17] Nelson P.P., Rath B.A., Fragkou P.C., Antalis E., Tsiodras S., Skevaki C. (2020). Current and Future Point-of-Care Tests for Emerging and New Respiratory Viruses and Future Perspectives. Front. Cell. Infect. Microbiol..

[18] Tai C.-S., Jian M.-J., Lin T.-H., Chung H.-Y., Chang C.-K., Perng C.-L., Hsieh P.-S., Shang H.-S. (2025). Analytical performance evaluation of a multiplex real-time RT-PCR kit for simultaneous detection of SARS-CoV-2, influenza A/B, and RSV. PeerJ.

[19] Schober T., Wong K., DeLisle G., Caya C., Brendish N.J., Clark T.W., Dendukuri N., Doan Q., Fontela P.S., Gore G.C. (2024). Clinical Outcomes of Rapid Respiratory Virus Testing in Emergency Departments: A Systematic Review and Meta-Analysis. JAMA Intern. Med..

[20] Lim H.-J., Lee J.-Y., Baek Y.-H., Park M.-Y., Youm D.-J., Kim I., Kim M.-J., Choi J., Sohn Y.-H., Park J.-E. (2023). Evaluation of Multiplex Rapid Antigen Tests for the Simultaneous Detection of SARS-CoV-2 and Influenza A/B Viruses. Biomedicines.

[21] (2020). Biological Evaluation of Medical Devices—Part 1: Evaluation and Testing Within a Risk Management Process.

[22] Meier K. (2007). Guidance for Industry and FDA Staff—Statistical Guidance on Reporting Results from Studies Evaluating Diagnostic Tests.

[23] McHugh M.L. (2012). Interrater reliability: The kappa statistic. Biochem. Med..

[24] Lee S. Guide to ROC and AUC for Statistical Analysis. https://www.numberanalytics.com/blog/roc-auc-guide-statistical-analysis.

[25] Chekol M.T., Sugerman D., Tayachew A., Mekuria Z., Tesfay N., Alemu A., Gashu A., Shura W., Gonta M., Agune A. (2025). Clinical and epidemiological characteristics of influenza and SARS-CoV-2 virus among patients with acute febrile illness in selected sites of Ethiopia 2021–2022. Front. Public Health.

[26] Dulo B., Hinsene G., Mannekulih E. (2024). Viral etiology of respiratory infections among patients at Adama Hospital Medical College, a facility-based surveillance site in Oromia, Ethiopia. medRxiv.

[27] CDC (2025). Multiplex Assays Authorized for Simultaneous Detection of Influenza Viruses and SARS-CoV-2 by FDA. Influenza (Flu). https://www.cdc.gov/flu/hcp/testing-methods/flu-covid19-detection.html.

[28] Basile K., Kok J., Dwyer D.E. (2017). Point-of-care diagnostics for respiratory viral infections. Expert. Rev. Mol. Diagn..

[29] Batra R., Blandford E., Kulasegaran-Shylini R., Futschik M.E., Bown A., Catton M., Conti-Frith H., Alexandridou A., Gill R., Milroy C. (2025). Multiplex lateral flow test sensitivity and specificity in detecting influenza, A, B and SARS-CoV-2 in adult patients in a UK emergency department. Emerg. Med. J..

[30] Carlton H.C., Savović J., Dawson S., Mitchelmore P.J., Elwenspoek M.M.C. (2021). Novel point-of-care biomarker combination tests to differentiate acute bacterial from viral respiratory tract infections to guide antibiotic prescribing: A systematic review. Clin. Microbiol. Infect..

[31] Rosenblatt K.P., Romeu H., Romeu C., Granger E. (2024). Performance evaluation of a SARS-CoV-2 and influenza A/B combo rapid antigen test. Front. Mol. Biosci..

[32] Bayart J.-L., Gillot C., Dogné J.-M., Roussel G., Verbelen V., Favresse J., Douxfils J. (2023). Clinical performance evaluation of the Fluorecare^®^ SARS-CoV-2 & Influenza A/B & RSV rapid antigen combo test in symptomatic individuals. J. Clin. Virol..

[33] Aboagye F.T., Annison L., Hackman H.K., Acquah M.E., Ashong Y., Owusu-Frimpong I., Egyam B.C., Annison S., Osei-Adjei G., Antwi-Baffour S. (2024). Comparative evaluation of RT-PCR and antigen-based rapid diagnostic tests (Ag-RDTs) for SARS-CoV-2 detection: Performance, variant specificity, and clinical implications. Microbiol. Spectr..

[34] Widyasari K., Kim S., Kim S., Lim C.S. (2022). Performance Evaluation of STANDARD Q COVID/FLU Ag Combo for Detection of SARS-CoV-2 and Influenza A/B. Diagnostics.

[35] Franck K.T., Schneider U.V., Ma C.M.G., Knudsen D., Lisby G. (2020). Evaluation of immuview RSV antigen test (SSI siagnostica) and BinaxNOW RSV card (alere) for rapid detection of respiratory syncytial virus in retrospectively and prospectively collected respiratory samples. J. Med Virol..

[36] Cantais A., Mory O., Plat A., Giraud A., Pozzetto B., Pillet S. (2019). Analytical performances of the BD VeritorTM System for the detection of respiratory syncytial virus and influenzaviruses A and B when used at bedside in the pediatric emergency department. J. Virol. Methods.

[37] Zhu M., Chen L., Cao J., Cai J., Huang S., Wang H., He H., Chen Z., Huang R., Ye H. (2025). Clinical application of Myxovirus resistance protein A as a diagnostic biomarker to differentiate viral and bacterial respiratory infections in pediatric patients. Front. Immunol..

[38] Jung J.-A., Wi P.-H., Kim H. (2020). Comparisons of clinical characteristics between Respiratory Syncytial Virus A and B infection. J. Allergy Clin. Immunol..

[39] van der Zalm M.M., Sam-Agudu N.A., Verhagen L.M. (2024). Respiratory adenovirus infections in children: A focus on Africa. Curr. Opin. Pediatr..

