# Supplementary material for: Evaluation of Flu A/B, SARS-CoV-2, and RSV Antigen Combo Rapid Test in Hospitalized Children Under Two Years of Age
Source: Diagnostics (Basel). 2026 Mar 11;16(6):830. doi: 10.3390/diagnostics16060830 (PMC13025871; doi:10.3390/diagnostics16060830)
Supplement: Supplementary file 1 [file diagnostics-16-00830-s001.zip › diagnostics-4005096-supplementary.pdf]

Supplementary Materials

Retesting of Samples with Invalid Allplex rRT-qPCR results

During Allplex rRT-qPCR testing, we observed that samples (n = 11) placed in the peripheral wells of 96-well plates yielded invalid results. We suspected edge effect due to evaporation and retested them in interior wells. Remarkably, 10 of the 11 samples produced valid results upon retesting, and all but one were subsequently confirmed as positive. For a sample that continued to produce invalid results, we performed a fresh RNA extraction from the original specimen and repeated the assay using an interior well. Despite these efforts, the result remained invalid. We suspect that PCR inhibitors may have been present in this sample, interfering with amplification.

Our observations indicate that edge effects (possibly due to evaporation) may have contributed to the initial invalid results seen in RNA samples placed in edge wells. Based on this experience, we recommend that a sample yielding an invalid result in the edge well needs to be retested.

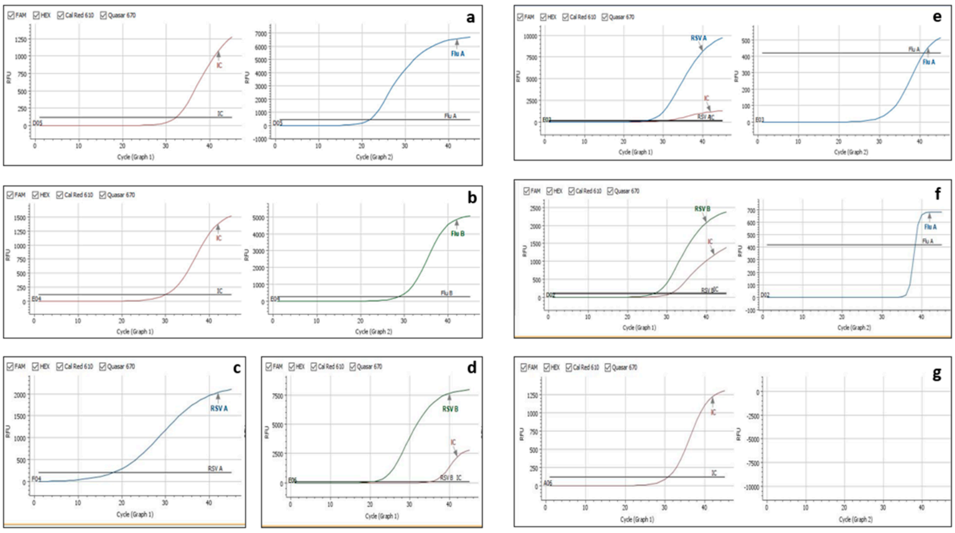

Figure S1: rRT-qPCR graph showing Ct values for selected specimen samples. (a), positive for Flu A, (b) positive Flu B, (c) positive RSVA, (d) positive RSV B; (e) mixed infection with RSV B and Flu A; (f) mixed RSV A and Flu A; and (g) tested negative for the targeted viruses. Abbreviations: rRT-qPCR, real-time reverse transcriptase quantitative polymerase chain reaction; Flu A, influenza A virus; Flu B, influenza B virus; RSV A, respiratory syncytial virus A; RSV B, respiratory syncytial virus B.

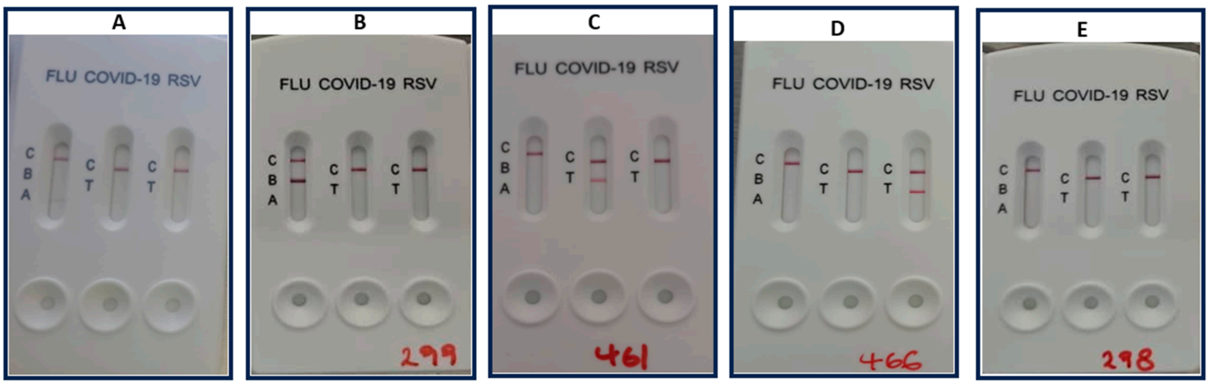

Figure S2: Photographs of the ML Ag Combo Rapid Test cassettes: A) Positive for influenza A (Flu A); (B) Positive for influenza B (Flu B); C) Positive for COVID-19; D) Positive for RSV; (E) Negative for all targeted viruses. Each cassette is divided into three columns—FLU, COVID-19, and RSV—each containing a corresponding control (C) line. The test includes four test (T) lines: two for FLU (lines A and B), one for COVID-19, and one for RSV. Results appear within 15 minutes following the application of an NPS sample mixed with the extraction buffer. For COVID-19 and RSV, a visible color band on both the T and C lines indicates a valid positive result. For influenza detection, a color band at line A, along with the C line, indicates a positive result for Flu A, while a color band at line B with the C line, indicates a positive result for Flu B. A single visible color band at the C line, with no accompanying test line, indicates a negative result for all targeted viruses.

Table S1: Sensitivity of Pooling of eight RNA samples with different proportions of known positive and negative RNA samples and their corresponding Ct values.

Pooled RNA samples	Ct value
rRT-qPCR[1] Pos (n =1) + PCR Neg (n = 7)	37.80
rRT-qPCR Pos (n= 2) + PCR Neg (n = 6)	37.58
rRT-qPCR Pos (n = 3) + PCR Neg (n = 5)	37.4
rRT-qPCR Pos (n =4) + PCR Neg (n = 4)	37.22

[1] Abbreviation: rRT-qPCR, real-time reverse transcriptase quantitative polymerase chain reaction; Pos, positive; Neg, negative; n, number of samples; Ct, cycle threshold.

Table S2: Two-by-Two Table for Test comparison between ML Ag Combo Rapid Test and Allplex rRT-qPCR.

**ML Ag Combo RDT**	**Allplex rRT-qPCR**		**Total**
RSV*	TP* =145	FP =5	150
	FN = 16	TN =304	320
Flu (A+B)	TP = 6	FP = 0	6
	FN = 14	TN = 450	464

* Abbreviations: TP, true plosive; FN, False negative; FP, False positive; TN, True negative
